# Difference between Right and Left Facial Surface Electromyography in Healthy People

**DOI:** 10.1155/2018/4069530

**Published:** 2018-08-16

**Authors:** Bo-Hyun Kim, Kyeong Han Kim, Lak-Hyung Kim, Jong-Uk Kim, Tae-Han Yook

**Affiliations:** ^1^Department of Acupuncture & Moxibustion Medicine, College of Korean Medicine, Woosuk University, Republic of Korea; ^2^Department of Preventive Medicine, College of Korean Medicine, Woosuk University, Republic of Korea; ^3^Department of Neuropsychiatry, College of Korean Medicine, Woosuk University, Republic of Korea

## Abstract

**Introduction:**

The study was to see whether there were differences in values of facial surface electromyography in subjects of good heath by muscles, age, and sex.

**Methods:**

It draws ratio between lower value and higher value (R-LV/HV) and asymmetry index (AI), based on root mean square (RMS) from measurement of facial surface electromyography (sEMG) in 154 people of healthy people (male:female = 70:84) aging between more than 20 and less than 70.

**Results:**

For R-LV/HV, it averages 81.70±14.60% on frontalis muscle, 73.74±19.12% on zygomaticus muscle, and 79.72±14.77% on orbicularis oris muscle. With analysis of the AI average was 10.87±10.14% on frontalis muscle, 16.71±14.79% on zygomaticus muscle, and 12.10±10.05% on orbicularis oris muscle. Both values were statistically significant in three parts of muscles as shown. Both of R-LV/HV and AI show no statistically significant difference on age and sex (p>0.05).

**Conclusions:**

It could provide basic data for the future diagnosis of facial nerve palsy patients by measuring facial sEMG values for healthy people.

## 1. Introduction

Facial paralysis has a major symptom of atonia paralysis on the facial muscle and was a disease with accompanying symptoms, such as decreased taste, hearing impairment, saliva secretion, and tear reduction [1]. Various hypotheses for causes of facial paralysis include viral infection, ischemic vascular disease-causing paralysis, vascular disorders due to diabetes, multiple neuritis, autoimmune disease, and cold exposure, but no hypothesis yet provides a clear explanation as to what causes facial paralysis [2]. It has been reported that 20-30 persons every 100,000 in population annually experience facial paralysis [3]. As of 2015, there were 8,511 cases of facial paralysis reported in Korea [4].

Diagnostic techniques for patients with facial paralysis include House-Brackmann scale, Yanagihara grading system, and Sunnybrook facial grading system, which were performed on naked eyes [5, 6], and digital infrared thermal imaging, or DITI, electroneurography (ENoG), nerve excitability test (NET) or electromyography (EMG), and surface electromyography (sEMG) were utilized as diagnostic instruments [7].

Among other things sEMG was a kind of EMG diagnostic instrument that quantitatively measures electric signals for muscle movements. In general, EMG was measuring the electromyogram of a single muscle by inserting a needle, while sEMG was a noninvasive mechanism using surficial electrodes and was advantageous in conducting the overall assessment of facial movements, not just movements of a single muscle. Because of its strength, sEMG has been widely used as an assessing instrument of facial paralysis and was an index that has significance in determining facial states and degree of recovery with ratio between lower value and higher value(R-LV/HV), or asymmetry index (AI) [8].

Although there have been many studies on sEMG of patents with facial paralysis, less studies have been reported on whether the indexes were different by sex, age, or parts of the body. In some study [9], there was a difference in facial sEMG values according to sex, but there was a limit of 40 small subjects. This study was to measure facial surface sEMG in subjects of good health aging between 20s and 60s to see any difference in the values depending on sex, age, or parts of the body.

## 2. Methods

### 2.1. Participants

The study mobilizes people aging older than 20 and less than 70 between September 13, 2016, and March 1, 2017. Prospective participants in the study were asked if they have the existing disease or have been administered with related medications through the basic examination and preliminary medical examination. Participants who were not subject to exclusion were selected as the final subjects. A total of 154 people wish to participate in the study and no subjects fall within exclusion. The final subjects include 154 people, 70 males and 84 females. Exclusion criteria are as follows.

1. Persons with anamnesis of stroke
2. Persons currently suffering from diseases associated with facial paralysis or with anamnesis of such diseases
3. Persons with facial disease or body disease that could affect other facial electromyography
4. Persons who have been given medications or experienced activity within one week that will affect measurement of sEMG
5. Persons having discomfort with facial muscle movements due to plastic surgery or facial operation
6. Persons who may have a displacement because they continuously use facial muscles in occupation (such as performer of brass instrument)
7. Persons with facial asymmetry of Grade 2 on House-Brackmann scale through naked eye assessment
8. Other cases of exclusion a researcher would determine inadequate

### 2.2. Study Implementation

#### 2.2.1. Medical Device

For sEMG implementation, a four-channel adopting electromyography system QEMG-4 XL (manufactured by Laxtha Co. Ltd., Korea) was used, while QEMG-4 XL (version1.0 Neuromedi Inc.) was used for measurement. For electrode sensor, AM530 active electromyography system (manufactured by Laxtha Co. Ltd., Korea) was used.

#### 2.2.2. R-LV/HV

A higher value from the left and right measurements in the total of 154 subjects was set on numerator and a lower value regarding as denominator. The formula is as follows.

(1) $$\begin{array}{c} Ratio\left(\;\%\;\right)=\frac{\text{EMG (low value side)}}{\text{EMG (High Value side)}}\times 100 \end{array}$$

#### 2.2.3. AI

AI is difference in the values divided by the sum of the values. In the study, the difference value obtained after deducting R-LV/HV, among root mean square (RMS) on the left and right measurements, was then divided by the sum of RMS values on the left and right parts to obtain AI. Higher AI means significant difference in RMS values on the left and right muscles. The formula is as follows.

(2) Asymmetry  Index%=EMG  high  value  side−EMG  (low  value  side)EMG  high  value  side−EMG  (low  value  side)×100

### 2.3. Measurement

The placement of electrode was made in parallel to muscular fibers of frontalis muscle, zygomaticus muscle, and orbicularis oris muscle. A first electrode was placed on the left, while a second electrode was place on the right. To eliminate factors reducing any skin resistance to the sEMG measuring signals, the measurement site was cleaned with medical alcohol cotton and its surfaces were to be completely dried before the electrode was placed. The real measurement was conducted when a subject fully learned how to move after preliminary measurement. The measurement of sEMG uses signal processing of root mean square (RMS). A relaxation time for one-time electromyography signal measurement was set on five seconds and tension time on three seconds. Gain index was between the ranges of -1463 and 1463. Each test was repeated with three measurements and the average measurement of the three measurements was used as a measurement value. Subjects were required to take a 10-minute rest and then return to the measurement of sEMG by having a disposable electrode placed on acupuncture points of frontalis muscle, zygomaticus muscle, and orbicularis oris muscle after being introduced how to move muscles at each acupuncture point.

#### 2.3.1. Frontalis Muscle (Yangbaek (GB14))

**Figure 1:**
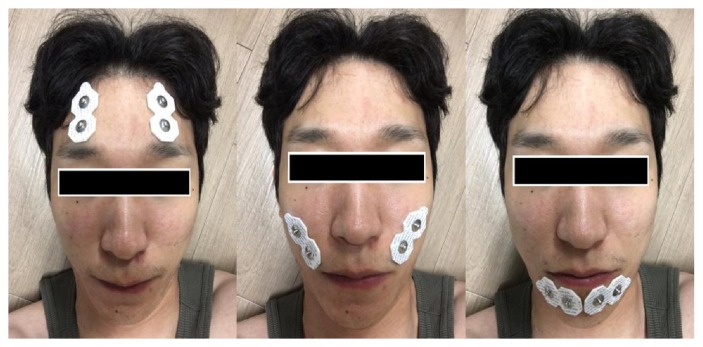
Attachment site of frontalis muscle, zygomaticus muscle, and orbicularis oris muscle.

The acupuncture point of* Yangbaek *(GB14) is located directly above pupil by a finger joint from the eyebrow [8]. For movements of frontalis muscle and* Yangbaek *(GB14), a subject is required to move the eyebrows to form wrinkles on his forehead (Figure 1).

#### 2.3.2. Zygomaticus Muscle (Gwonyo (SI18))

The acupuncture point of* Gwonyo *(SI18) is located sunken at ends of Yegol below Myeonpigol [8]. For movements of zygomaticus muscle and* Gwonyo *(SI18), a subject is required to pull angular upward and outside (Figure 1).

#### 2.3.3. Orbicularis Oris Muscle (Seungjang (CV24))

The acupuncture point of* Seungjang *(CV24) is located sunken at edges of the lips [8]. Orbicularis oris muscle is located by a finger joint from both sides of Seungjang (CV24). For movements of orbicularis oris muscle, a subject is required to pucker lips forward to hold out (Figure 1).

### 2.4. Statistical Analysis

For statistics of research results, SPSS Statistics 22.0 version 64 bit edition (IBM, USA) was used and all the measurements were indicated in mean±SD. For sex comparison, paired t-test was conducted and one-way analysis of variance (ANOVA) followed by a post hoc Scheffe test was used to compare muscle and age. When* p*-value was less than 0.05, this was interpreted to have a statistically significance, and all the values were rounded up from the third decimal place.

### 2.5. Ethics

The study was approved by Institutional Review Board, IRB of Jeonju Oriental Hospital in affiliation with Woosuk University (No. WSOH IRB 1610-06).

## 3. Results

### 3.1. Sociodemographic Characteristics

The gathered group of males and female were in their 20s and 60s. For age distribution of the people gathered, 31 people were in the range of between older than 20 and younger than 30, 29 people were in the range of between older than 30 and younger than 40, 37 people were in the range of between older than 40 and younger than 50, 29 people were in the range of between older than 50 and younger than 60 and 28 people were in the range of between older than 60 and younger than 70. The average height of male was 171.56±5.29cm and average weight was 72.34±8.74kg. The average height of female was 160.25±5.22cm and average weight was 58.17±8.80 (Table 1).

### 3.2. Difference in R-LV/HV and AI between Muscles

The total of 154 subjects on the three parts have R-LV/HV and AI were measured (Table 2). For R-LV/HV, it averages on 81.70±14.60% on frontalis muscle, 73.74±19.12% on zygomaticus muscle, and 79.72±14.77% on orbicularis oris muscle. When they were compared using one-way ANOVA, R-LV/HV on each part shows statistically significant difference (p<0.001). Frontalis muscle value was higher than zygomaticus muscle value and orbicularis oris muscle value was higher than zygomaticus muscle value. However, it was no significant difference between frontalis muscle value and orbicularis oris muscle value.

For AI, it averages on 10.87±10.14% on frontalis muscle, 16.71±14.79% on zygomaticus muscle, and 12.10±10.05% on orbicularis oris muscle (Table 2). When they were compared using one-way ANOVA, the asymmetry index on each part shows statistically significant difference (*p<*0.001). Frontalis muscle value was lower than zygomaticus muscle value and orbicularis oris muscle value was lower than zygomaticus muscle value. However, there was no significant difference between frontalis muscle value and orbicularis oris muscle value.

### 3.3. Difference in R-LV/HV and AI between Ages

For R-LV/HV, it has its highest of 84.05±15.54% in 50s on frontalis muscle and its lowest of 78.75±19.01% in 30s. It has its highest of 79.76±17.87% in 30s on zygomaticus muscle and its lowest of 69.13±18.03% in 60s. It has its highest of 81.26±12.63% in 30s on orbicular oris muscle and its lowest of 76.52±16.33% in 20s. There was no significant difference between age in R-LV/HV (p>0.05) (Table 3).

For AI, it has its highest of 13.22±13.11% in 30s on frontalis muscle and its lowest of 9.17±7.64% in 20s. It has its highest of 19.88±16.14% in 60s on zygomaticus muscle and its lowest of 12.45±12.57% in 30s. It has its highest of 14.29±11.20% in 20s on orbicular oris muscle and its lowest of 10.88±8.19% in 30s. There was no significant difference between age in AI (p>0.05) (Table 3).

### 3.4. Difference in R-LV/HV and AI between Male and Female

For R-LV/HV, it averages on 82.37±15.29% in 70 males on frontalis muscle, 75.40±19.32% on zygomaticus muscle, and 80.00±15.50% on orbicularis oris muscle. It averages on 81.15±14.07% in 84 females on frontalis muscle, 72.35±18.96% on zygomaticus muscle, and 79.49±14.21% on orbicularis oris muscle. When they were compared using paired t-test, the R-LV/HV by sex shows no statistically significant difference on all the three parts (p>0.05) (Table 4).

For AI, it averages on 10.56±10.89% in 70 males on frontalis muscle, 15.62±14.86% on zygomaticus muscle, and 12.03±10.84% on orbicularis oris muscle. It averages on 11.13±9.53% in 84 females on frontalis muscle, 17.62±14.77% on zygomaticus muscle, and 12.16±9.40% on orbicularis oris muscle. When they were compared using paired t-test, the R-LV/HV by sex shows no statistically significant difference on all the three parts (p>0.05) (Table 4).

## 4. Discussion

Most widely used methods for assessing facial paralysis include House-Brackmann scale, Yanagihara grading system, and Sunnybrook facial grading system which were methods of assessment with facial movements in patients as well as utilization of diagnosis devices such as DITI, NET, ENoG, EMG, and sEMG [10].

Among these methods, sEMG was a test that measures action potential by attaching electrodes on surfaces of the skin. Muscle forms a figure of the body by being attached to skins and the skeletal system and supervises exercise of the whole body that moves the skeleton system. When muscle retraction occurs, this simultaneously triggers motor impulse signal on motor cortex in the brain which was continuously transmitted into nerves of each motor unit through motor neurons of the spinal cord [11, 12]. When these nerve impulses were brought to neuromuscular junction, this would cause an electric transmission along muscular fibers to sarcolemma in both directions, which was called motor unit action potential, or MUAP [13]. Electrodiagnosis was defined as capturing, amplifying, and recording the electric action in muscles and was based on measurements of occurrence, mobilization, and propagation of these action potentials to be displayed on screen. The electrodes of the sEMG were divided into pole electrodes and surface electrodes based on measuring sites and convenience, and the surface electrode was used to alleviate pains of subjects when the measurement was conducted [14]. Unlike other methods, the sEMG was regarded as a relatively simple procedure not requiring artificial electric stimulus or noninvasive stimulus and thus has a potential for popularity, especially for the facial parts because a patient shows no resistance to its use on them. A study on surficial sEMG was emerging as the new paradigm in the field of rehabilitation for muscular and skeletal disease. There have been brisk study efforts in overseas going on the sEMG, including types of electrode and location of placement [15, 16].

For literature review on related studies on sEMG conducted in Asian countries such as Korea, Japan, and China, there were less than 10 studies on the sEMG in each country as of 2012 and there was the only clinical literature on patients with facial paralysis accompanying coordination movements [17]. Although there have been attempts to interpret results of the sEMG test on patients with facial paralysis by associating with the naked eye test, it was insufficient to represent the sEMG test with the smaller number of samples of 21 people. Another ongoing study on the sEMG measurements of 20 males and 40 females with good health when they move their facial parts was designed to draw biological electric features of the local specimen with good health but shows its limitation in that it fails to provide no classification other than classification by males and females as well as with the smaller number of specimen [18, 19].

For sEMG analysis, the widely used statistical analysis was used. RMS was a method that analyzes amplitude of signal shown in sEMG. The analysis was available to measure the number of motor units activated and firing rate as it represents an increasing aspect of the signal amplitude when the muscle retracts and a decreasing aspect when muscle fatigue occurs [20]. It also has a significance in its utilization when R-LV/HV, Al determine conditions of the facial parts and degree of recovery. No report has been made as to whether the indexes of subjects with good health have a difference in sex, age, or parts of the body.

The authors in the study obtained R-LV/HV, Al from measurements of the facial surface sEMG in the subjects aged between 20s and 60s and with good health to clarify if the values have any difference in sex, age, or parts of the body. The study was based on a total of 154 males and females who aged between 20s and 60s and with good health gathered starting from September 13, 2016, to May 1, 2017. The selected subjects were required to take a 10-minute rest and introduced to learn how to move muscles of each acupuncture point to measure sEMG by placing a disposable electrode on acupuncture points of frontalis muscle, zygomaticus muscle, and orbicularis oris muscle. To measure orbicularis oris muscle, it selects a lower orbicularis oris muscle by a finger joint from both sides of Seungjanghyeol and this is based on results found in the study of Kim et al. [21] that orbicularis oris muscle has a higher measurement on the lower orbicularis oris muscle than on the higher orbicularis oris muscle.

The total of 154 subjects on the three parts have R-LV/HV and AI were measured. For R-LV/HV, it averages on 81.70±14.60% on frontalis muscle, 73.74±19.12% on zygomaticus muscle, and 79.72±14.77% on orbicularis oris muscle. When they were compared using one-way ANOVA, R-LV/HV on each part shows statistically significant difference. Frontalis muscle value was higher than zygomaticus muscle value and orbicularis oris muscle value was higher than zygomaticus muscle value. However, it was no significant difference between frontalis muscle value and orbicularis oris muscle value. For AI, it averages on 10.87±10.14% on frontalis muscle, 16.71±14.79% on zygomaticus muscle, and 12.10±10.05% on orbicularis oris muscle. When they were compared using one-way ANOVA, the asymmetry index on each part shows statistically significant difference. Frontalis muscle value was lower than zygomaticus muscle value and orbicularis oris muscle value was lower than zygomaticus muscle value. However, it was no significant difference between frontalis muscle value and orbicularis oris muscle value.

For R-LV/HV, it has its highest of 84.05±15.54% in 50s on frontalis muscle and its lowest of 78.75±19.01% in 30s. It has its highest of 79.76±17.87% in 30s on zygomaticus muscle and its lowest of 69.13±18.03% in 60s. It has its highest of 81.26±12.63% in 30s on orbicular oris muscle and its lowest of 76.52±16.33% in 20s. There was no significant difference between age in R-LV/HV. For AI, it has its highest of 13.22±13.11% in 30s on frontalis muscle and its lowest of 9.17±7.64% in 20s. It has its highest of 19.88±16.14% in 60s on zygomaticus muscle and its lowest of 12.45±12.57% in 30s. It has its highest of 14.29±11.20% in 20s on orbicular oris muscle and its lowest of 10.88±8.19% in 30s. There was no significant difference between age in AI.

For R-LV/HV, it averages on 82.37±15.29% in 70 males on frontalis muscle, 75.40±19.32% on zygomaticus muscle, and 80.00±15.50% on orbicularis oris muscle. It averages on 81.15±14.07% in 84 females on frontalis muscle, 72.35±18.96% on zygomaticus muscle, and 79.49±14.21% on orbicularis oris muscle. When they were compared using paired t-test, the R-LV/HV by sex shows no statistically significant difference on all the three parts. For AI, it averages on 10.56±10.89% in 70 males on frontalis muscle, 15.62±14.86% on zygomaticus muscle, and 12.03±10.84% on orbicularis oris muscle. It averages on 11.13±9.53% in 84 females on frontalis muscle, 17.62±14.77% on zygomaticus muscle, and 12.16±9.40% on orbicularis oris muscle. When they were compared using paired t-test, the R-LV/HV by sex shows no statistically significant difference on all the three parts.

As found in the results stated above, individual differences in RMS values of the sEMG in subjects of good health were large and the same large differences were found in range of standard deviation, while R-LV/HV or AI in subjects of good health shows relatively certain range of values. This comes down to the conclusion as stated in Lee et al. [19] that RMS values were not suitable for determining conditions of subjects, rather comparisons using R-LV/HVs and AI values can be more reasonable method to determine whether a subject has a normal condition or not. Based on the conclusion, the results in this study can be utilized to assess abnormality of facial muscles and determine whether they fall within the normality.

## 5. Conclusion

The study analyzes R-LV/HV and AI with measurements of surface electromyography or sEMG on acupunctures points such as frontalis muscle, zygomaticus muscle, and orbicularis oris in a total of 154 subjects with good health.

1. For R-LV/HV, it averages 81.70±14.60% on frontalis muscle, 73.74±19.12% on zygomaticus muscle, and 79.72±14.77% on orbicularis oris muscle. With analysis of the AI average was 10.87±10.14% on frontalis muscle, 16.71±14.79% on zygomaticus muscle, and 12.10±10.05% on orbicularis oris muscle.
2. R-LV/HV was significance in three parts of muscles (FM>ZM, ZM<OM, FM=OM). And AI also was significance difference in muscles (FM<ZM, ZM>OM, FM=OM)
3. Both of R-LV/HV and AI showed no statistically significant difference on age and sex.

In subjects of good health, no difference was found in terms of R-LV/HV and AI either by sex or by age. It was anticipated that the results in this study will be utilized to determine diagnosis, prognosis, and recovery in the future.

## Figures and Tables

**Table 1:** Demographic characteristics of 154 subjects.

Classification	Male	Female	Total
Age (person)			
Total	70	84	154
20s	14	17	31
30s	15	14	29
40s	14	23	37
50s	14	15	29
60s	13	15	28
height (cm)	171.56±5.29	160.25±5.22	165.39±7.70
weight (kg)	72.34±8.74	58.17±8.80	64.61±11.25

**Table 2:** Difference R-LV/HV and AI between muscles.

classification	FM	ZM	OM	F	p	Post Hoc
	Mean±SD (%)	Mean±SD (%)	Mean±SD (%)			
R-LV/HV	81.70±14.60	73.74±19.12	79.72±14.77	9.965	<.001	FM>ZM
						ZM<OM
						FM=OM
AI	10.87±10.14	16.71±14.79	12.10±10.05	10.365	<.001	FM<ZM
						ZM>OM
						FM=OM

*∗* FM: frontalis muscle; ZM: zygomaticus muscle; OM: orbicularis oris muscle.

**Table 3:** Average of R-LV/HV and AI between ages.

classification	20s	30s	40s	50s	60s	F	p
	Mean±SD (%)	Mean±SD (%)	Mean±SD (%)	Mean±SD (%)	Mean±SD (%)		
R-LV/HV							
FM	83.99±11.99	78.75±19.01	81.65±13.32	84.05±15.54	79.86±12.72	.781	.539
ZM	76.11±14.99	79.77±17.87	72.66±18.93	71.00±24.27	69.14±18.03	1.440	.224
OM	76.53±16.33	81.26±12.64	80.65±15.43	79.71±16.29	80.44±12.86	.487	.745
AI							
FM	9.18±7.65	13.23±13.12	10.76±9.31	9.61±11.71	11.76±8.35	.770	.546
ZM	14.41±10.34	12.46±12.57	17.32±14.05	19.58±19.34	19.89±16.15	1.413	.232
OM	14.30±11.20	10.89±8.19	11.60±10.75	12.28±11.38	11.40±8.17	.528	.715

*∗* FM: frontalis muscle; ZM: zygomaticus muscle; OM: orbicularis oris muscle.

**Table 4:** Average of each muscles in R-LV/HV and AI between male and female.

classification	Male (N=70)	Female (N=84)	Total (N=154)	t	p
	Mean±SD (%)	Mean±SD (%)	Mean±SD (%)		
R-LV/HV					
FM	82.37±15.29	81.15±14.07	81.70±14.60	.512	.610
ZM	75.40±19.32	72.35±18.96	73.74±19.12	.983	.327
OM	80.00±15.50	79.49±14.21	79.72±14.77	.213	.831
AI					
FM	10.56±10.89	11.13±9.53	10.87±10.14	-.348	.728
ZM	15.62±14.86	17.62±14.77	16.71±14.79	-.834	.406
OM	12.03±10.84	12.16±9.40	12.10±10.05	-.083	.934

*∗* FM: frontalis muscle; ZM: zygomaticus muscle; OM: orbicularis oris muscle.

## Data Availability

The data used to support the findings of this study may be released upon application to the IRB of Jeonju Oriental Hospital in affiliation with Woosuk University.
